# Genome Sequence of Ralstonia pseudosolanacearum SL1931, a Causal Phytopathogen of Bacterial Wilt Disease in Capsicum annuum and Nicotiana benthamiana

**DOI:** 10.1128/mra.00278-22

**Published:** 2022-06-07

**Authors:** Myoungjoo Riu, Choong-Min Ryu, Soohyun Lee, Seon-Woo Lee, Seungdon Lee, Sang-Keun Oh, Haeyoung Jeong

**Affiliations:** a Infectious Disease Research Center, KRIBB, Daejeon, South Korea; b Department of Applied Biology, College of Agriculture & Life Sciences, Chungnam National University, Daejeon, South Korea; c Department of Biosystems and Bioengineering Program, KRIBB School of Biotechnology, University of Science and Technology (UST), Daejeon, South Korea; d Department of Applied Biology, Dong-A University, Busan, South Korea; e Department of Agricultural Biology, National Institute of Agricultural Sciences, Wanju, South Korea; University of Arizona

## Abstract

Here, we report the genome sequence of Ralstonia pseudosolanacearum (R. solanacearum phylotype I) strain SL1931 (KACC10711), isolated from pepper (Capsicum annuum L.) stems; *R. solanacearum* is the causal pathogen of bacterial wilt. Strain SL1931 had a different type III effector profile than that of the reference genome strain GMI1000.

## ANNOUNCEMENT

Ralstonia solanacearum, which is the causal pathogen of bacterial wilt disease, infects more than 250 plant species, most of which belong to the Solanaceae family, including pepper, tobacco, tomato, and potato (1, 2). R. solanacearum invades the xylem vessels through plant root hairs and disseminates into the stem, where it multiplies and causes wilting by blocking water transport and producing excessive exopolysaccharides (3). Virulence and pathogenicity factors of R. solanacearum are known from previous studies (4, 5). Here, the genome sequence of Ralstonia pseudosolanacearum (phylotype I) race 1, biovar 3 strain SL1931 (KACC10711), is presented. *R. pseudosolanacearum* phylotype I strain GMI1000 was the first strain subjected to whole-genome analysis and therefore serves a reference genome. Strain GMI1000 is pathogenic to a model dicot plant, Arabidopsis thaliana (6, 7). On the other hand, *R. pseudosolanaceraum* strain SL1931 is not virulent to *A. thaliana* but causes severe disease symptoms in Nicotiana benthamiana.

Strain SL1931 was originally isolated from pepper stems in Seosan, Chungcheongnam-do, South Korea, in 1998. The strain was preserved as freeze-dried cultures in the Korean Agricultural Culture Collection (KACC), Wanju, South Korea. The freeze-dried cultures were rehydrated under laboratory conditions in KRIBB, South Korea. The cultures were stored in a 20% glycerol suspension at −80°C for long-term preservation and routine work. Strain SL1931 was cultured on Casamino Acids-peptone-glucose (CPG) agar medium at 30°C for 24 h for purity testing. A single colony was picked and cultured in CPG broth for 8 h. The 1% volume of seed culture was inoculated in CPG broth and cultured for 18 h. Then, genomic DNA (gDNA) of SL1931 was extracted using the Wizard Genomic DNA purification kit (Promega, Madison, WI, USA) according to the manufacturer’s protocol.

Library preparation and sequencing were carried using a PacBio RS II system (P6-C4 chemistry) at the Chun Lab (Seoul, South Korea). The sequencing library was constructed according to the manufacturer’s instructions for 20-kb template preparation using the PacBio DNA template prep kit 1.0 and BluePippin size selection system. A total of 111,636 reads (999.42 Mb) were produced with an average length of 8,952 bp and *N*_50_ of 12,217 bp, with an average read quality of 0.857. Default parameters were used for all software unless otherwise specified. *De novo* assembly and two additional rounds of polishing were performed with RS_HGAP_Assembly.2 and RS_Resequencing.1 protocols, respectively, using SMRT Analysis v2.3.0 (https://smrt-analysis.readthedocs.io/en/latest/SMRT-Analysis-Software-Installation-v2.3.0/). Polished sequences were circularized with Circlator v1.1.5 (8).

A chromosome (3,744,397 bp, 66.85% G+C) and a megaplasmid (2,075,606 bp, 66.85% G+C), along with two additional plasmids (50,417 bp, 61.76% G+C; 34,545 bp, 60.5% G+C), were obtained. Circular topologies for all four replicons were confirmed by Unicycler v0.4.8 (9). Genome completeness and contamination were 100.0% and 0%, respectively, based on quality assessment analysis by CheckM v1.1.3 (10). Two-way average nucleotide identity (ANI) with strain LMG 9673 (RefSeq GCF_919586305.1) was 95.95% based on calculations from the Kostas lab website (http://enve-omics.ce.gatech.edu/ani/). Among all *Ralstonia* strain genome sequences available to date (490 GenBank entries as of 14 March 2022), strain Pe_1 (GCA_011420365.1, accession no. SAMN13503698), which is one of the 30 South Korean isolates studied under project no. PRJNA593908, was found to be closest to that of SL1931 (99.98% ANI) as calculated by fastANI v1.32 (11).

The NCBI Prokaryotic Genome Annotation Pipeline (PGAP) (12) was used for automatic sequence annotation. Type III effector genes were predicted using the Ralsto T3E database (13). The results showed that the type III effectors RipAL and RipBP were only present in the SL1931 strain and absent in GMI1000. Twenty-three effectors in strain GMI1000 were lacking in strain SL1931, which indicated different host specificity between the two strains, although both were classified within the same phylotype I.

### Data availability.

The complete sequences, including the chromosome and three plasmids, were deposited in the DDBJ/ENA/GenBank databases under accession no. CP093535 for the chromosome and CP093536, CP093537, and CP093538 for the plasmids. Project data are available under BioProject accession no. PRJNA814415. The PacBio raw sequencing reads were submitted to the NCBI Sequence Read Archive under accession no. SRR18296424.

## References

[1] Safni I, Cleenwerck I, De Vos P, Fegan M, Sly L, Kappler U. 2014. Polyphasic taxonomic revision of the Ralstonia solanacearum species complex: proposal to emend the descriptions of Ralstonia solanacearum and Ralstonia syzygii and reclassify current R. syzygii strains as Ralstonia syzygii subsp. syzygii subsp. nov., R. solanacearum phylotype IV strains as Ralstonia syzygii subsp. indonesiensis subsp. nov., banana blood disease bacterium strains as Ralstonia syzygii subsp. celebesensis subsp. nov. and R. solanacearum phylotype I and III strains as Ralstonia pseudosolanacearum sp. nov. Int J Syst Evol Microbiol 64:3087–3103. doi:10.1099/ijs.0.066712-0. 24944341

[2] Xu C, Zhong L, Huang Z, Li C, Lian J, Zheng X, Liang Y. 2022. Real-time monitoring of Ralstonia solanacearum infection progress in tomato and Arabidopsis using bioluminescence imaging technology. Plant Methods 18:7. doi:10.1186/s13007-022-00841-x. 35033123 PMC8761306

[3] Schell MA. 2000. Control of virulence and pathogenicity genes of Ralstonia solanacearum by an elaborate sensory network. Annu Rev Phytopathol 38:263–292. doi:10.1146/annurev.phyto.38.1.263. 11701844

[4] de Pedro-Jové R, Puigvert M, Sebastià P, Macho AP, Monteiro JS, Coll NS, Setúbal JC, Valls M. 2021. Dynamic expression of Ralstonia solanacearum virulence factors and metabolism-controlling genes during plant infection. BMC Genomics 22:170. doi:10.1186/s12864-021-07457-w. 33750302 PMC7941725

[5] Shen F, Yin W, Song S, Zhang Z, Ye P, Zhang Y, Zhou J, He F, Li P, Deng Y. 2020. Ralstonia solanacearum promotes pathogenicity by utilizing l-glutamic acid from host plants. Mol Plant Pathol 21:1099–1110. doi:10.1111/mpp.12963. 32599676 PMC7368120

[6] Salanoubat M, Genin S, Artiguenave F, Gouzy J, Mangenot S, Arlat M, Billault A, Brottier P, Camus JC, Cattolico L, Chandler M, Choisne N, Claudel-Renard C, Cunnac S, Demange N, Gaspin C, Lavie M, Moisan A, Robert C, Saurin W, Schiex T, Siguier P, Thébault P, Whalen M, Wincker P, Levy M, Weissenbach J, Boucher CA. 2002. Genome sequence of the plant pathogen Ralstonia solanacearum. Nature 415:497–502. doi:10.1038/415497a. 11823852

[7] Liu Y, Tang Y, Qin X, Yang L, Jiang G, Li S, Ding W. 2017. Genome sequencing of Ralstonia solanacearum CQPS-1, a phylotype I strain collected from a highland area with continuous cropping of tobacco. Front Microbiol 8:974. doi:10.3389/fmicb.2017.00974. 28620361 PMC5449461

[8] Hunt M, Silva ND, Otto TD, Parkhill J, Keane JA, Harris SR. 2015. Circlator: automated circularization of genome assemblies using long sequencing reads. Genome Biol 16:294. doi:10.1186/s13059-015-0849-0. 26714481 PMC4699355

[9] Wick RR, Judd LM, Gorrie CL, Holt KE. 2017. Unicycler: resolving bacterial genome assemblies from short and long sequencing reads. PLoS Comput Biol 13:e1005595. doi:10.1371/journal.pcbi.1005595. 28594827 PMC5481147

[10] Parks DH, Imelfort M, Skennerton CT, Hugenholtz P, Tyson GW. 2015. CheckM: assessing the quality of microbial genomes recovered from isolates, single cells, and metagenomes. Genome Res 25:1043–1055. doi:10.1101/gr.186072.114. 25977477 PMC4484387

[11] Jain C, Rodriguez-R LM, Phillippy AM, Konstantinidis KT, Aluru S. 2018. High throughput ANI analysis of 90K prokaryotic genomes reveals clear species boundaries. Nat Commun 9:5114. doi:10.1038/s41467-018-07641-9. 30504855 PMC6269478

[12] Tatusova T, DiCuccio M, Badretdin A, Chetvernin V, Nawrocki EP, Zaslavsky L, Lomsadze A, Pruitt KD, Borodovsky M, Ostell J. 2016. NCBI prokaryotic genome annotation pipeline. Nucleic Acids Res 44:6614–6624. doi:10.1093/nar/gkw569. 27342282 PMC5001611

[13] Peeters N, Carrère S, Anisimova M, Plener L, Cazalé A-C, Genin S. 2013. Repertoire, unified nomenclature and evolution of the type III effector gene set in the Ralstonia solanacearum species complex. BMC Genomics 14:859. doi:10.1186/1471-2164-14-859. 24314259 PMC3878972

